# Ten years of follow-up data in psoriatic arthritis: results based on standardized monitoring of patients in an ordinary outpatient clinic in southern Norway

**DOI:** 10.1186/s13075-018-1659-z

**Published:** 2018-08-02

**Authors:** Glenn Haugeberg, Brigitte Michelsen, Stig Tengesdal, Inger Johanne Widding Hansen, Andreas Diamantopoulos, Arthur Kavanaugh

**Affiliations:** 1 Division of Rheumatology, Department of Medicine, Hospital of Southern Norway Trust, Servicebox 416, 4604 Kristiansand, Norway; 2 Department of Neuroscience, Division of Rheumatology, Norwegian University of Science and Technology, Trondheim, Norway; 3 Department of Rheumatology, Martina Hansens Hospital, Bærum, Norway; 4 Division of Rheumatology, Allergy, and Immunology, School of Medicine, University of California, San Diego, USA

**Keywords:** Psoriatic arthritis, Clinical outcome, Disease activity, Patient-reported outcome measures, Treat to target, Real life registries

## Abstract

**Background:**

Over the last decade, a treat-to-target (T2T) strategy has been recommended for psoriatic arthritis (PsA) and new treatment options have become available. There is a lack of data on PsA regarding any changes that may have occurred over these past years. Thus, the main aim of this study was to look for changes in clinical disease status and treatment in a PsA outpatient clinic population monitored over the period 2008 to 2017.

**Methods:**

Annual data collection included demographic data, laboratory (erythrocyte sedimentation rate (ESR) and C-reactive protein (CRP)) and clinic measures of disease activity (e.g., 28 and 32 joint count Disease Activity Score (DAS28), Clinical Disease Activity Index (CDAI), and modified Disease Activity index for Psoriatic arthritis (DAPSA)), evaluator’s global assessment, and patient-reported outcomes (PROs), including for example measures of physical function, pain, and patient global assessment. Disease-modifying antirheumatic drug (DMARD) use was also registered.

**Results:**

In the PsA outpatient clinic population over the 10-year period (annual mean number of patients, 331) the mean (standard deviation) age was 58.4 (12.4) years, disease duration was 9.6 (7.9) years, 49.4% were female, and 17.6% were current smokers. From 2008 to 2017, no statistically significant increase in remission rates was seen for DAPSA (13.5% and 22.0%) or Boolean remission (6.6% and 8.9%), whereas a statistically significant increase was seen for DAS28-ESR (36.8% and 50.6%) and CDAI (20.0% and 29.6%), but not for the last 5 years (DAS28-ESR, 42.3% and 50.6%; CDAI, 27.9% and 29.6%). Furthermore, over the 10-year period no significant improvement for PROs and no significant change in the use of synthetic (annual mean 53.0%) and biologic DMARDs (annual mean 29.9%) was found.

**Conclusion:**

Our data suggest that even in the biologic treatment era there is an unmet need for treating PsA patients to target remission. New treatment options and the development of more feasible and valid outcome measures for use in a T2T strategy in ordinary clinical practice may in the future to further improve clinical outcomes in PsA.

## Background

In the new millennium, new treatment strategies (early intervention and treat-to-target (T2T)) have become the new standard of clinical follow-up for patients with chronic inflammatory joint disorders [1]. The T2T strategy was first recommended for use in rheumatoid arthritis (RA) where its significant impact on improved clinical outcome has been convincingly documented [2, 3]. Encouraged by the evidence in RA, an international task force in 2012 recommended the T2T strategy also be used in spondyloarthritis (SpA), including psoriatic arthritis (PsA). The recommendations, however, were mainly based on expert opinion [4]. With new data available strengthening these recommendations, a revised updated version was published in 2018 [5].

In the new millennium, new treatment options with a broad range of targeted modes of action have become available for treatment of chronic inflammatory joint disorders, including PsA [6].

Most data available in the literature are based on selected patient groups included in, for example, registries or clinical studies. Data reflecting unselected outpatient clinic cohorts with data obtained from patients monitored using clinical outcome measures are rare.

We have previously published data on 10-year change in disease status and treatment for RA based on standardized monitoring in an ordinary outpatient clinic in southern Norway [7]. In that study, we documented the dramatic improvement in clinical outcomes and prognosis for RA that took place in a Norwegian outpatient clinic in the period from 2004 to 2013 [7]. To our knowledge, no longitudinal observational study data exist on changes in clinical outcomes and treatment for PsA outpatients reflecting an entire PsA outpatient clinic cohort monitored with standardized outcome measures as part of ordinary clinical practice.

Thus, the aim of this study was to explore the long-term changes in clinical disease status and treatment in Norwegian PsA outpatients, monitored as part of standard clinical care in the era of biologic treatment.

## Methods

### Patients and data collection

The outpatient rheumatology clinic serves a population of approximately 290,000 inhabitants living in the two most southern counties in Norway. In the same geographic era there are also two private practicing rheumatologists.

At the outpatient clinic, the standard for monitoring patients with recommended outcome measures was first introduced in 2003 for RA patients. In 2005, the computer software program GoTreatIT® Rheuma (www.diagraphit.com) was implemented at the outpatient clinic, facilitating patient monitoring with selected outcome measures. During 2007, regularly monitoring of not only RA but also PsA patients was implemented as part of standard clinical care. For RA patients, no specific protocol for tight control or any specific treatment protocol was used [7]. Treatment and follow-up visits were based on the treating doctor’s judgment performed in accordance with national recommendations and, after 2007, also in accordance with the Norwegian tender system for prescription of biologic disease-modifying antirheumatic drugs (bDMARDs).

The same standard outcome measures used for monitoring RA patients at the outpatient clinic were also applied to monitor the PsA patients. Patient-reported outcome (PRO) measures included the Modified Health Assessment Questionnaire (MHAQ) assessing physical function [8], visual analog scales (VAS; 0–100 mm) used to report pain, joint pain, fatigue, and patient global assessment (PGA). Morning stiffness was reported in 15-min units. Standard assessment did not include assessment of skin, nails, entheses, or dactylitis.

Standardized 28 and 32 swollen and tender joint counts were performed by rheumatologists or by trained nurses. The 32-joint count included the 28-joint count plus standardized joint count of ankles and metatarsophalangeal joints (MTP) joints, both scored from 0–2 (the MTP joints were scored as one joint). Laboratory markers of inflammation included C-reactive protein (CRP; mg/L) and erythrocyte sedimentation rate (ESR; mm/h). The 28-joint composite Disease Activity Score (DAS) with ESR (DAS28-ESR) [9], the Clinical Disease Activity Index (CDAI) [10], and a modified version of the Disease Activity index for Psoriatic arthritis (DAPSA) including a 32-joint count instead of the original 66/68-joint count was calculated [11]. The evaluator’s (trained nurse or rheumatologist) global assessment (EGA) of disease activity was reported on a VAS (0–100 mm). Data on rheumatoid factor (RF) were also recorded.

We used the cut-offs for DAS28-ESR, CDAI, and DAPSA to define remission, low disease activity, moderate disease activity, and high disease activity [10, 12, 13]. We also applied the suggested DAS28-ESR cut-off from Salaffi et al. of 2.4 instead of 2.6 for defining remission in PsA [14]. Furthermore, the Boolean remission criteria in accordance with the new American College of Rheumatology/European League Against Rheumatism (ACR/EULAR) guidelines for remission were tested [15].

Previous and current treatment use was systematically registered and updated at all visits, including use of prednisolone, synthetic DMARDs (sDMARDs), and bDMARDs. Demographic data collected included gender, age, weight, height, body mass index (BMI), smoking status, disease duration, and work status. From 2010 onwards, self-reported height and weight, smoking status, years of education, and work status were included as part of the standard routine with the use of the computer program. In the analysis we only included PsA patients who fulfilled the Classification for Psoriatic Arthritis criteria (CASPAR) and who were 18 years or older [16]. Data retrieved from the computer were based on data from the last annual patient visit for each year.

### Statistical analysis

Continuous variables are presented as mean and standard deviation (SD). Categorical variables are presented as numbers and percentages. To look for a change in variables and associations over the 10-year period and the last 5 years of this period we used linear regression for continuous variables and the Chi-square test for categorical variables. A *p* value of < 0.05 was taken to be statistically significant.

## Results

The number of PsA patients with at least one annual visit during the follow-up period ranged from 106 patients in 2008 to a maximum of 412 in 2014, with a mean annual number of 331 patients. From 2008 to 2010, the number of PsA patients increased from 106 to 318 and thereafter stabilized at a mean annual number of 367 patients in the subsequent years.

Data for gender, age, BMI, years of education, full time job status, smoking status, disease duration, and RF status are shown in Table 1. Apart from age and disease duration, no statistically significant differences during follow-up were seen, as shown in Table 1. Over the 10-year period, mean annual proportions were: females 49.4%; patients with a full-time job (age < 65 years) 35.8%; current smokers 17.6%; and RF-positive patients 4.8%. Mean (SD) annual values for the period were: age, 58.4 (12.4) years; BMI 27.6 (4.7) kg/m^2^; education 12.5 (3.6) years; and disease duration 9.6 (7.9) years.

**Table 1:** Clinical and demographic data for our study sample

Year (patients)	Females, *n* (%)	Age (years)	BMI (kg/m^2^)	Education (years)	Full-time job if aged < 65 years, *n* (%)	Current smoker, *n* (%)	Disease duration (years)	RF-positive, *n* (%)
2008 (*n* = 106)	52 (49.1%) [100%]	59.5 (13.5) [100%]	NA	NA	NA	NA	7.3 (8.0) [100%]	4 (7.1%) [52.8%]
2009 (*n* = 268)	140 (52.2%) [100%]	60.3 (11.8) [100%]	NA	NA	NA	NA	8.2 (7.4) [100%]	10 (6.7%) 150 [56.0%]
2010 (*n* = 318)	158 (49.7%) [100%]	60.5 (12.1) [100%]	27.4 (4.8) [91.8%]	12.3 (3.5) [94.0%]	73 (39.2%) 194^a^ [95.9%]	59 (19.6%) [94.7%]	8.3 (7.2) [100%]	14 (6.8%) [64.5%]
2011 (*n* = 365)	181 (49.6%) [100%]	59.4 (12.5) [100%]	27.6 (4.8) [97.3%]	12.3 (3.7) [88.5%]	84 (40.2%) 233^a^ [89.7%]	71 (21.8%) [89.3%]	8.9 (7.4) [100%]	15 (5.6%) [73.4%]
2012 (*n* = 377)	179 (47.5%) [100%]	58.6 (12.3) [100%]	27.4 (4.8) [96.6%]	12.3 (3.7) [97.3%]	86 (35.5%) 250^a^ [96.8%]	70 (19.1%) [97.1%]	9.2 (7.4) [100%]	17 (5.8%) [78.2%]
2013 (*n* = 397)	197 (49.6%) [100%]	58.8 (12.3) [100%]	27.6 (4.4) [98.0%]	12.4 (3.6) [98.2%]	93 (35.6%) 264^a^ [98.9%]	69 (17.6%) [98.5%]	9.7 (7.9) [100%]	17 (5.1%) 335 [84.4%]
2014 (*n* = 412)	208 (50.5%) [100%]	58.3 (12.3) [100%]	27.7 (4.6) [96.8%]	12.6 (3.4) [97.1%]	107 (39.1%) 282^a^ [97.2%]	71 (17.7%) [97.3%]	9.8 (7.9) [100%]	13 (3.9%) [81.1%]
2015 (*n* = 384)	197 (51.3%) [100%]	56.8 (12.6) [100%]	27.7 (4.7) [98.2%]	12.5 (3.6) [97.7%]	90 (32.8%) 280^a^ [97.9%]	60 (15.9%) [98.2%]	10.3 (8.1) [100%]	10 (3.3%) [79.9%]
2016 (*n* = 340)	165 (48.5%) [100%]	57.1 (12.6) [100%]	27.7 (5.0) [97.4%]	12.6 (3.5) [96.8%]	68 (29.2%) 239^a^ [97.5%]	51 (15.4%) [97.6%]	10.6 (8.3) [100%]	9 (3.2%) [82.4%]
2017 (*n* = 341)	158 (46.3%) [100%]	56.0 (12.3) [100%]	28.0 (4.8) [97.9%]	12.9 (3.4) [97.1%]	87 (36.0%) 249^a^ [97.2%]	47 (14.1%) [97.7%]	11.3 (8.5) [100%]	10 (3.6%) [80.6%]
Mean for the period	49.4%	58.4 (12.4)	27.6 (4.7)	12.5 (3.6)	35.8%	17.6%	9.6 (7.9)	4.8%
*P* value^b^ 2008–2017	0.94	< 0.001	NA	NA	NA	NA	< 0.001	0.41
*P* value^b^ 2013–2017	0.71	0.001	0.38	0.11	0.34	0.64	0.003	0.74

Values are shown as mean (standard deviation) unless otherwise indicated

Percentages within square brackets represent patients with available data

*BMI* body mass index, *RF* rheumatoid factor, *NA* not available

^a^Numbers of patients aged > 65 years

^b^χ^2^ test for categorical and linear regression for continuous variables was used to test for differences during follow-up

Table 2 shows the measures of disease activity displayed for each year in the 10-year period. A statistically significant improvement in all disease activity measures was seen for the 10-year period; however, for the last 5 years of follow-up (2013–2017) a statistically significant improvement was only seen for ESR and for DAS28-ESR, but not for CRP, joint count, CDAI, modified DAPSA, or EGA.

**Table 2:** Measures of disease activity for each year from 2008 to 2017 for patients with psoriatic arthritis monitored with outcome measures in an ordinary outpatient clinic

Year (patients)	ESR (mm/h)	CRP (mg/L)	28 tender joint count	28 swollen joint count	DAS28-ESR	CDAI	DAPSA	EGA (VAS, mm)
2008 (*n* = 106)	15.8 (13.1) [80.2%]	7.6 (10.2) [86.8%]	3.3 (5.6) [92.5%]	1.5 (2.0) [92.5%]	3.32 (1.49) [53.8%]	10.09 (8.98) [51.9%]	13.0 (9.9) [49.1%]	14.5 (14.5) [84.9%]
2009 (*n* = 268)	15.3 (14.0) [71.3%]	5.8 (9.2) [79.9%]	2.5 (4.6) [96.6%]	1.2 (2.2) [96.6%]	3.05 (1.29) [64.6%]	9.25 (7.97) [80.2%]	12.4 (8.6) [67.5%]	13.2 (12.6) [90.3%]
2010 (*n* = 318)	15.8 (14.5) [77.7%]	5.4 (8.9) [83.3%]	1.7 (3.5) [96.9%]	0.8 (1.7) [96.9%]	2.79 (1.13) [74.2%]	7.35 (6.34) [89.9%]	11.1 (7.9) [78.6%]	11.6 (11.2) [93.7%]
2011 (*n* = 365)	14.1 (12.4) [77.8%]	4.9 (11.1) [82.2%]	1.6 (3.6) [93.4%]	0.6 (1.6) [93.4%]	2.64 (1.06) [75.9%]	6.94 (6.30) [87.9%]	10.6 (8.0) [77.5%]	11.6 (11.8) [90.4%]
2012 (*n* = 377)	12.6 (9.9) [71.4%]	4.2 (6.8) [78.0%]	1.3 (3.2) [93.9%]	0.5 (1.3) [93.9%]	2.52 (1.08) [66.3%]	6.45 (5.90) [87.8%]	9.9 (7.3) [71.6%]	9.7 (11.2) [92.0%]
2013 (*n* = 397)	14.2 (13.1) [77,6%]	4.5 (9.0) [82.9%]	1.5 (3.3) [94.0%]	0.4 (1.1) [94.0%]	2.66 (1.07) [75.1%]	6.60 (5.54) [87.7%]	10.3 (7.1) [78.3%]	10.0 (10.8) [91.9%]
2014 (*n* = 412)	14.6 (13.5) [78.6%]	4.7 (6.9) [83.3%]	1.3 (3.1) [96.6%]	0.4 (1.3) [96.6%]	2.56 (1.08) [74.0%]	6.27 (5.58) [90.0%]	9.8 (7.2) [76.0%]	8.4 (9.4) [95.4%]
2015 (*n* = 384)	14.0 (13.8) [78.9%]	4.5 (9.8) [86.7%]	1.4 (3.3) [97.4%]	0.4 (1.4) [97.4%]	2.59 (1.12) [74.2%]	6.66 (6.11) [91.1%]	10.4 (7.9) [78.9%]	9.0 (9.5) [96.4%]
2016 (*n* = 340)	13.2 (12.8) [77.9%]	5.2 (9.4) [87.1%]	1.3 (3.0) [93.8%]	0.4 (1.0) [93.8%]	2.46 (1.03) [68.5%]	6.39 (5.17) [82.6%]	10.0 (6.8) [74.1%]	9.2 (9.9) [91.5%]
2017 (*n* = 341)	10.9 (11.2) [59.8%]	4.1 (7.4) [69.2%]	1.6 (3.6) [78.9%]	0.6 (1.5) [78.9%]	2.46 (1.12) [49.3%]	6.76 (6.08) [68.3%]	10.4 (7.6) [54.5%]	8.6 (11.4) [80.1%]
Mean for the period	14.0 (12.9)	4.9 (8.9)	1.6 (3.6)	0.6 (1.5)	2.64 (1.13)	6.93 (6.19)	10.5 (7.7)	10.2 (11.0)
*P* value^a^ 2008–2017	< 0.001	0.01	< 0.001	< 0.001	< 0.001	< 0.001	0.001	< 0.001
*P* value^a^ 2013–2017	0.004	0.92	0.94	0.28	0.03	0.71	0.48	0.32

Values are shown as mean (standard deviation)

The percentages within square brackets represent patients with available data

*CDAI* Clinical Disease Activity Index, *CRP* C-reactive protein, *DAPSA* Disease Activity index for Psoriatic arthritis, *DAS* Disease Activity Score, *EGA* evaluator’s global assessment, *ESR* erythrocyte sedimentation rate, *VAS* visual analog scale

^a^Linear regression was used to test for differences during follow-up

Comparing the two joint counts, the swollen/tender 32-joint count was a mean of 0.1/0.3 higher than the 28-joint count (annual detailed data for 32-joint counts are not shown).

**Fig. 1:**
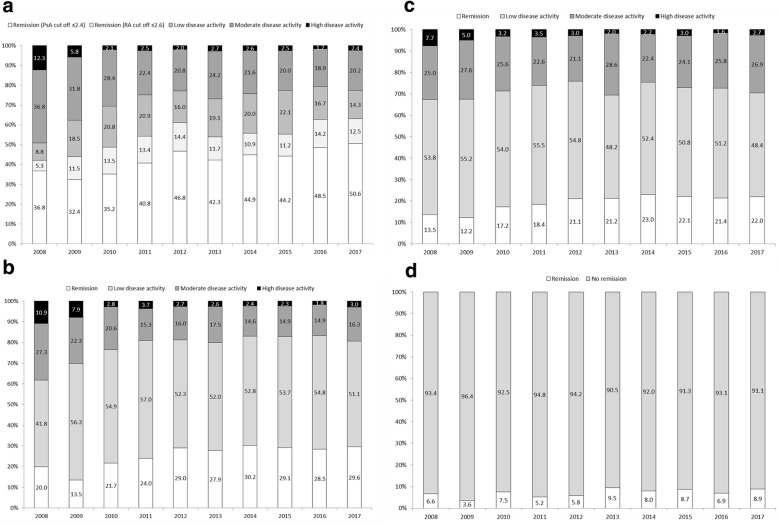
Percentage of patients with psoriatic arthritis (PsA) in remission and with low, moderate, and high disease activity for each year in the 10-year period from 2008 to 2017, **a** defined according to cut-offs for the Disease Activity Score in 28 joints [12], **b** defined according to cut-offs for the Clinical Disease Activity Index [10], **c** defined according to cut-offs for the Disease Activity index for Psoriatic arthritis [13], and **d** percentages of patients in remission as defined by Boolean criteria [15]. RA rheumatoid arthritis

As shown in Fig. 1, the proportion of patients in remission was dependent on the composite measures used. The lowest remission rates were found when applying the ACR/EULAR Boolean criteria (range 3.6% to 9.5%) and the modified DAPSA criteria (range 12.2% to 23.0%). The highest remission rates were shown for the DAS28-ESR criteria (range 42.1% to 63.1%) and CDAI criteria (range 13.5% to 30.2%), both developed and validated for use in RA. When using the DAS28-ESR cut off ≤ 2.4, as recommended by Salaffi et al. to be applied when used in PsA [14], the remission rates were lower (range 32.4% to 50.6%); however, these were still significantly higher than for DAPSA and the ACR/EULAR Boolean remission criteria. A significant increase over the 10-year period was only seen for DAS28-ESR and CDAI remission and not for Boolean and DAPSA remission. For the last 5 years of follow-up, no significant change in remission rates occurred for any of the remission criteria.

As shown in Table 3, no significant improvement was seen for either the 10-year period or the last 5 years of follow-up for PRO measures. In contrast, in the last 5 years of follow-up, a small but statistically significant deterioration was seen for MHAQ, pain, joint pain, and morning stiffness, but not for fatigue or PGA.

**Table 3:** Patient-reported outcome measures for each year from 2008 to 2017 for patients with psoriatic arthritis monitored in an ordinary outpatient clinic

Year (patients)	MHAQ (0–3)	Pain (VAS, mm)	Joint pain (VAS, mm)	Fatigue (VAS, mm)	Morning stiffness (h)	PGA (VAS, mm)
2008 (*n* = 106)	0.59 (0.53) 59.4%]	37.5 (24.6) [56.6%]	41.1 (25.2) 36 [34.0%]	44.5 (32.4) [58.5%]	1.24 (1.48) [58.5%]	41.3 (26.0) [58.5%]
2009 (*n* = 268)	0.46 (0.44) [85.8%]	37.5 (22.1) [84.3%]	38.1 (23.0) [84.3%]	47.0 (30.2) [84.7%]	0.93 (1.05) [85.1%]	40.3 (24.4) [87.3%]
2010 (*n* = 318)	0.45 (0.43) [93.4%]	37.4 (23.4) [93.1%]	36.2 (23.5) [91.8%]	43.2 (31.1) [92.1%]	0.90 (1.15) [92.8%]	37.6 (23.3) [93.7%]
2011 (*n* = 365)	0.44 (0.42) [89.9%]	36.1 (24.2) [89.6%]	35.4 (24.1) [89.3%]	44.9 (30.6) [89.3%]	0.90 (1.21) [90.4%]	37.1 (23.9) [91.5%]
2012 (*n* = 377)	0.44 (0.43) [89.7%]	36.2 (24.5) [89.7%]	35.6 (24.7) [89.7%]	42.9 (30.2) [89.7%]	0.93 (1.27) [89.1%]	37.6 (25.6) [91.0%]
2013 (*n* = 397)	0.42 (0.40) [90.1%]	33.9 (25.5) [90.2%]	33.8 (23.7) [90.7%]	42.6 (31.0) [90.4%]	0.87 (1.15) [90.7%]	37.4 (26.2) [90.9%]
2014 (*n* = 412)	0.45 (0.44) [90.0%]	35.8 (24.7) [89.1%]	35.0 (24.7) [87.1%]	42.7 (31.3) [87.1%]	0.89 (1.14) [86.7%]	37.9 (26.5) [91.3%]
2015 (*n* = 384)	0.47 (0.43) [91.4%]	38.4 (26.5) [91.1%]	36.2 (26.6) [90.1%]	45.5 (32.6) [90.4%]	0.97 (1.27) [89.8%]	40.1 (28.2) [93.0%]
2016 (*n* = 340)	0.51 (0.46) [87.6%]	38.8 (26.8) [86.2%]	38.3 (26.5) [87.4%]	45.2 (32.2) [87.6%]	1.06 (1.36) [86.8%]	40.6 (27.8) [87.9%]
2017 (*n* = 341)	0.51 (0.45) [89.4%]	39.8 (26.4) [89.4%]	38.7 (26.3) [89.4%]	45.1 (32.7) [89.1%]	1.03 (1.27) [89.7%]	40.6 (27.9) [90.6%]
Mean for the period	0.46 (0.44)	37.0 (24.8)	36.3 (24.9)	44.2 (31.3)	0.95 (1.22)	38.8 (26.0)
*P* value^a^ 2008–2017	0.11	0.10	0.44	0.90	0.26	0.18
*P* value^a^ 2013–2017	0.002	0.001	0.003	0.16	0.025	0.06

Values are shown as mean (standard deviation)

The percentages within square brackets represent patients with available data

*MHAQ* Modified Health Assessment Questionnaire, *PGA* patient’s global assessment, *VAS* visual analog scale

^a^ Linear regression was used to test for differences during follow-up

In Table 4 the proportions of PsA patients treated with prednisolone, sDMARD, and bDMARD monotherapy or combination therapy are shown. The proportion of patients on no treatment declined significantly over the 10-year period from approximately 30% in 2008 to approximately 20% in 2017. The proportion of PsA patients using prednisolone (annual mean 14.9%, range 12.6% to 22.6%), sDMARDs (annual mean 53.0%, range 50.7% to 56.3%), and bDMARDs (annual mean 29.9%, range 23.5% to 32.8%) remained stable overall over the 10-year period. The use of sDMARDs was dominated by the use of methotrexate, and bDMARDs by the use of tumor necrosis factor (TNF) inhibitors. The annual mean percentage of PsA patients using the different sDMARDs was 38.5% for methotrexate, 11.2% for leflunomide, 2.4% for sulfasalazine, and 0.9% for other sDMARDs. Only a few PsA patients were treated with bDMARDs with modes of action other than TNF inhibitors: ustekinumab and sekukinumab. However, the use of sekukinumab, which was introduced in Norway in 2016, for PsA-treated patients increased from 0.6% in 2016 to 2.6% in 2017.

**Table 4:** Treatment is displayed for each year in the 10-year period from 2008 to 2017 for patients with psoriatic arthritis monitored with outcome measures in an ordinary outpatient clinic

Treatment	2008 (*n* = 106)	2009 (*n* = 268)	2010 (*n* = 318)	2011 (*n* = 365)	2012 (*n* = 377)	2013 (*n* = 397)	2014 (*n* = 412)	2015 (*n* = 384)	2016 (*n* = 340)	2017 (*n* = 341)	Annual mean	*P* value^a^2008–17 and 2013–17
No treatment^b^ (%)	29 (27.4)	92 (34.3)	96 (30.2)	115 (31.5)	115 (30.5)	112 (28.2)	113 (27.4)	102 (26.6)	72 (21.2)	65 (19.1)	27.5%	< 0.001 0.011
bDMARD (%)	32 (30.2)	63 (23.5)	88 (27.7)	99 (27.1)	113 (30.0)	120 (30.2)	129 (31.3)	125 (32.6)	107 (31.5)	112 (32.8)	29.9%	0.29 0.94
TNF inhibitor (%)	31 (29.2)	59 (22.0)	83 (26.1)	94 (25.8)	110 (29.2)	115 (29.0)	121 (29.4)	118 (30.7)	99 (29.1)	100 (29.3)	28.1%	0.43 0.99
Non-TNF inhibitors (%)	1 (0.9)	4 (1.5)	5 (1.6)	5 (1.4)	3 (0.8)	5 (1.3)	8 (1.9)	7 (1.8)	8 (2.4)	12 (3.5)	1.8%	0.32 0.30
sDMARD (%)	59 (55.7)	136 (50.7)	168 (52.8)	188 (51.5)	192 (50.9)	208 (52.4)	215 (52.2)	206 (53.6)	188 (55.3)	192 (56.3)	53.0%	0.89 0.75
One sDMARD (%)	59 (55.7)	136 (50.7)	168 (52.8)	188 (51.5)	191 (50.7)	208 (52.4)	214 (51.9)	205 (53.4)	188 (55.3)	191 (56.0)	52.8%	0.96 0.87
Two sDMARDs (%)	0 (0)	0 (0)	0 (0)	0 (0)	1 (0.3)	0 (0)	1 (0.2)	1 (0.3)	0 (0)	1 (0.3)	0.1%	–
bDMARD and sDMARD (%)	20 (18.9)	36 (13.4)	51 (16.0)	52 (14.2)	61 (16.2)	66 (16.6)	68 (16.5)	68 (17.7)	52 (15.3)	53 (15.5)	15.9%	0.91 0.91
TNF inhibitor and sDMARD (%)	20 (18.9)	35 (13.1)	50 (15.7)	52 (14.2)	61 (16.2)	64 (16.1)	65 (15.8)	64 (16.7)	49 (14.4)	47 (13.8)	15.3%	0.89 0.81
Prednisolone (%)	24 (22.6)	39 (14.6)	49 (15.4)	52 (14.2)	49 (13.0)	56 (14.1)	52 (12.6)	54 (14.1)	59 (17.4)	60 (17.6)	14.9%	0.22 0.23
Prednisolone and sDMARD (%)	16 (15.1)	23 (8.6)	29 (9.1)	32 (8.8)	26 (6.9)	28 (7.1)	25 (6.1)	28 (7.3)	31 (9.1)	31 (9.1)	8.1%	0.19 0.44
Prednisolone, sDMARD, and bDMARD (%)	4 (3.8)	8 (3.0)	9 (2.8)	7 (1.9)	5 (1.3)	9 (2.3)	5 (1.2)	5 (1.3)	5 (1.5)	5 (1.5)	1.9%	0.47 0.77
Biologic DMARDs												
Adalimumab (%)	11 (10.4)	31 (11.6)	41 (12.9)	44 (12.1)	46 (12.2)	48 (12.1)	44 (10.7)	41 (10.7)	25 (7.4)	28 (8.2)	10.9%	0.33 0.18
Certolizumab (%)	0 (0)	0 (0)	0 (0)	1 (0.3)	1 (0.3)	1 (0.3)	12 (2.9)	16 (4.2)	18 (5.3)	15 (4.4)	1.9%	< 0.001 0.001
Etanercept (%)^c^	8 (7.5)	20 (7.5)	26 (8.2)	37 (10.1)	44 (11.7)	33 (8.3)	34 (8.3)	31 (8.1)	30 (8.8)	34 (10.0)	9.0%	0.69 0.90
Golimumab (%)	0 (0)	0 (0)	9 (2.8)	5 (1.4)	7 (1.9)	23 (5.8)	19 (4.6)	12 (3.1)	11 (3.2)	8 (2.3)	2.8%	< 0.001 0.11
Infliximab (%)^d^	12 (11.3)	8 (3.0)	7 (2.2)	7 (1.9)	12 (3.2)	10 (2.5)	12 (2.9)	19 (4.9)	15 (4.4)	15 (4.4)	3.5%	0.001 0.31
Abatacept (%)	1 (0.9)	4 (1.5)	4 (1.3)	2 (0.5)	2 (0.5)	4 (1.0)	2 (0.5)	2 (0.5)	1 (0.3)	1 (0.3)	0.7%	0.65 0.66
Ustekinumab (%)	0 (0)	0 (0)	0 (0)	1 (0.3)	0 (0)	1 (0.3)	6 (1.5)	4 (1.0)	5 (1.5)	2 (0.6)	0.6%	0.022 0.33
Sekukinumab (%)	0 (0)	0 (0)	0 (0)	0 (0)	0 (0)	0 (0)	0 (0)	0 (0)	2 (0.6)	9 (2.6)	0.3%	< 0.001< 0.001
Other biologics (%)	0 (0)	0 (0)	1 (0.3)	2 (0.5)	1 (0.3)	0 (0)	0 (0)	1 (0.3)	0 (0)	0 (0)	0.2%	0.55 0.42
Synthetic DMARDs (%)												
Leflunomide	6 (5.7)	19 (7.1)	27 (8.5)	37 (10.1)	48 (12.7)	53 (13.4)	58 (14.1)	49 (12.8)	36 (10.6)	37 (10.9)	11.2%	0.037 0.52
Methotrexate	48 (45.3)	106 (39.6)	124 (39.0)	134 (36.7)	130 (34.5)	144 (36.3)	151 (36.7)	147 (38.3)	141 (41.5)	149 (43.7)	38.5%	0.22 0.18
Sulfasalazine	4 (3.8)	8 (3.0)	13 (4.1)	13 (3.6)	11 (2.9)	7 (1.8)	4 (1.0)	8 (2.1)	8 (2.4)	5 (1.5)	2.4%	0.16 0.62
Other synthetic DMARDs^e^	1 (0.9)	3 (1.1)	4 (1.3)	4 (1.1)	4 (1.1)	4 (1.0)	3 (0.7)	3 (0.8)	3 (0.9)	2 (0.6)	0.9%	1.00 0.98
Ever users												
Biologic DMARDs (%)	41 (38.7)	74 (27.6)	106 (33.3)	123 (33.7)	138 (36.6)	152 (38.3)	165 (40.0)	171 (44.5)	150 (44.1)	157 (46.0)	38.6%	< 0.001 0.16
TNF inhibitor (%)	41 (38.7)	74 (27.6)	105 (33.0)	122 (33.4)	137 (36.3)	150 (37.8)	164 (39.8)	168 (43.8)	147 (43.2)	156 (45.7)	38.2%	< 0.001 0.17
Non-TNF inhibitors (%)	2 (1.9)	5 (1.9)	8 (2.5)	7 (1.9)	7 (1.9)	11 (2.8)	16 (3.9)	17 (4.4)	21 (6.2)	20 (5.9)	3.4%	0.004 0.15

Data are presented as *n* (%)

*bDMARD* biologic disease-modifying antirheumatic drug, *sDMARD* synthetic disease-modifying antirheumatic drug, *TNF* tumor necrosis factor

^a^χ^2^ test used to test for differences during follow-up for the period 2008–2017 and 2013–2017

^b^No prednisolone, bDMARD or sDMARD

^c^Includes the originator and SB4

^d^Includes the originator and the biosimilar CT-P13

^e^Includes hydroxychloquine, auranofin, azathioprine

The use of a combination of sDMARDs and bDMARDs was also stable over the years (annual mean 15.9%, range 13.4% to 18.9%). Detailed information on use of specific sDMARDs and bDMARDs is shown in Table 4.

Over the 10-year period, significantly more PsA patients had been ever-users of bDMARDs, ranging from 27.6% in 2009 to 46.0% in 2017; however, for the last 5 years no significant increase for ever-use of bDMARDs was found.

## Discussion

In our PsA outpatient clinic population for the 2008 to 2017 period, a statistically significant improvement in measures reflecting disease activity was observed. However, for the last 5 years of follow-up, a statistically significant improvement was only seen for ESR and DAS28-ESR and not for CRP, 28-joint count, CDAI, EGA, or DAPSA. Furthermore, no significant improvement in physical functioning or patient perception of, for example, fatigue, pain, and morning stiffness was found. A statistically significant increase in remission rates was only found for the entire period for DAS28-ESR (range 32.4% to 50.6%) and CDAI (range 13.5% to 30.2%), but not for ACR/EULAR Boolean (range 3.6% to 9.5%) and DAPSA (range 12.2% to 23.0%) remission or for DAS28-ESR (range 42.3 to 50.6%) and CDAI (range 27.9 to 30.2%) remission for the last 5 years of follow-up.

The remission rates observed in our PsA cohort seems to be lower than that found in our RA outpatient clinic population for the period 2004 to 2013, where remission rates increased significantly not only for DAS28-ESR but also for ACR/EULAR Boolean remission [7]. For comparison with the period 2008 to 2013, the remission rates in the reported RA patients increased from 24.7% to 55.5% for DAS28-ESR and from 6.8% to 17.7% for Boolean remission, whereas the remission rates in our PsA patients increased from 32.4% to 46.8% for DAS28-ESR and from 3.5% to 9.5% for the ACR/EULAR Boolean. However, this comparison should be interpreted with caution. Our data confirm the results from others that DAS28-ESR and CDAI (developed for use in RA) overestimates the remission rates in PsA compared with DAPSA [17], and this occurs even when using the recommended DAS28-ESR remission cut-off ≤ 2.4 from Salaffi et al. to define remission in PsA [14]. When aiming for a less stringent T2T goal, including low disease activity, the differences between DAS28-ESR, CDAI, and DAPSA was less striking, as shown in Fig. 1.

The reduced access over the last years to modern treatment options with different modes of action other than TNF inhibitors in PsA compared with RA patients may partly explain the impression of a less significant improvement in clinical outcomes seen in our study. This is illustrated by 13.5% of RA patients from our outpatient clinic using non-TNF inhibitor bDMARDs in 2013, whereas the figures for our PsA patients were 1.3% in 2013 and 3.5% in 2017 [7]. The new drugs with mode of actions other than TNF inhibition used in our study included interleukin (IL)-12/23 inhibition (ustekinumab) and IL-17A inhibition (sekukinumab). The Janus kinase inhibitor tofacitinib has also been shown to be effective in treating PsA and is expected to soon reach the Norwegian market [18]. The number of PsA patients receiving no treatment (neither prednisolone nor DMARDs) in our study was in the range 20–30%. These figures are high compared with those we reported in RA, where approximately 10% of RA patients received no prednisolone or DMARD treatment [7]. One reason for this may be that a large proportion of PsA patients present as mono- or oligoarthritis. For example, in a Norwegian PsA study, 5.8% had mono- and 22.9% had oligoarthritis [19]. These patients may be less likely than the polyarthritis PsA patients to be treated with DMARDs and this may partly explain the rather high proportion of PsA patients receiving no treatment in our study.

According to EULAR recommendations, it is recommended that RA patients in need of bDMARDs (especially TNF inhibitors) be treated in combination with sDMARDs because of the superior efficacy compared with monotherapy using either sDMARDs or bDMARDs [20]. In PsA there is no such strong evidence for combination treatment, as is also reflected in the EULAR recommendations for the management of PsA [21]. In our study, approximately 50% of the TNF inhibitor-treated PsA patients were also on sDMARDs. In comparison, approximately 75% of the TNF inhibitor-treated RA patients in 2013 were treated in combination with sDMARDs [7]. The rather high rate of bDMARD-treated PsA patients on concomitant sDMARDs may not only be explained by the physician’s belief of a better effect using the combination, but could also be explained by a physician’s concern about immunogenicity using bDMARD monotherapy without combination with, for example, methotrexate [22].

Challenges related to the heterogeneity of the PsA disease are reflected in the use of outcome measures in our study but are also shown by the different recommendations on use of outcome measures; some favor the use of unidimensional composite scores (e.g., DAPSA) focusing on articular inflammation [23], and some favor the use of multidimensional scores [24] including the different disease domains [5]. This heterogeneity of the PsA disease is therefore a major challenge when assessing the burden of disease both in the clinic and in research. Despite all these challenges, the concept of remission as a treatment goal in PsA is gaining more and more acceptance among rheumatologists. Higher scores for PROs (e.g., pain and fatigue) have been reported in PsA compared with RA, in spite of lower swollen joint counts [25]. The higher scores for pain reported in PsA patients seen in the literature may be explained by the inflammatory involvement of entheses and the presence of dactylitis which were not assessed in our study.

Despite recommendations for treating PsA patients to target, and agreement among rheumatologist favoring this approach, there is a discrepancy with what occurs in real life. This issue was explored in the study by Gvozdenovic and colleagues [26]. In their study, 83% agreed that composite measures should be recorded regularly in RA patients; however, the real-life data revealed that in only 54% of the patients were composite scores actually recorded at ≥ 50% of the patient visits. There is reason to believe that this is also the case for PsA and, due to the heterogeneity of the PsA disease, the figures may be even worse in PsA than in RA. One strategy to improve the use of outcome measures in clinical practice could be the use of dedicated and trained nurse practitioners and physician assistants [27]. Further systematic education in outpatient clinics, for example implementing a learning collaborative, may also improve the adherence to a T2T strategy [28]. Another strategy could be developing patient self-assessment tools. For example, for the psoriasis area and severity index (PASI), a validated patient self-administered psoriasis score has been developed called the self-administered psoriasis area and severity index (SAPASI) [29, 30]. The implementation of outcome measures in daily clinical practice can also be facilitated by the use of computer technology as, for example, in our study and in DANBIO [31, 32].

Our study has several limitations, including the use of outcome measures developed and validated for use in RA, no examination of the skin, nails, entheses or dactylitis, and the use of the 32-joint count and not the 66/68-swollen and tender joint count in the calculation of DAPSA [11]. This most likely has underestimated the DAPSA score in our PsA outpatients since the use of a reduced joint count has been shown to miss a significant number of PsA patients with active disease [33]. However, the mean difference between 28- and 32-joint count was minor in our study (mean 0.1 for swollen and 0.3 for tender joints). We also emphasize that the composite scores of DAS28 and CDAI with their cut-offs to define disease status and the Boolean remission criteria have not been validated for use in PsA. It is, however, a paradox that even recent approval of novel therapies for PsA have been based on clinical trials using primary endpoints derived from RA.

Other limitations of our study include assessment bias through examination by various physicians at various time points, and missing data, which may affect the internal validity of the results. The generalizability of the results may also have been affected by patient recruitment being only from one center. However, there is no obvious reason to believe that the examined PsA outpatient clinic cohort is different from other outpatient clinic cohorts in Norway. Despite all the limitations in this study, the long-term monitoring of PsA patients reflecting an entire PsA outpatient clinic population is in itself rather unique, and our data may thus be of interest both for clinicians and researchers in contributing to an increased understanding of the disease burden in PsA in our time.

## Conclusions

The overall interpretation of our results is that there is still an unmet need in treating PsA patients to target, even in the era of biologic treatment. There is also an urgent need to develop, validate, and agree on feasible outcome measures to be used in ordinary clinical care, capturing the heterogenic expression of the PsA disease. However, in the meantime, our study should encourage clinicians to implement the use of available and feasible outcome measures in ordinary clinical care, for example DAPSA, to improve patient outcome.

## Abbreviations

ACR/EULAR: American College of Rheumatology/European League Against Rheumatism
bDMARD: Biologic disease-modifying antirheumatic drug
BMI: Body mass index
CDAI: Clinical Disease Activity Index
CRP: C-reactive protein
DAPSA: Disease Activity index for Psoriatic arthritis
DAS: Disease Activity Score
DMARD: Disease-modifying antirheumatic drug
EGA: Evaluator’s global assessment
ESR: Erythrocyte sedimentation rate
MHAQ: Modified Health Assessment Questionnaire
MTP: Metatarsophalangeal
PGA: Patient global assessment
PRO: Patient-reported outcome
PsA: Psoriatic arthritis
RA: Rheumatoid arthritis
RF: Rheumatoid factor
SD: Standard deviation
sDMARD: Synthetic disease-modifying antirheumatic drug
T2T: Treat-to-target
TNF: Tumor necrosis factor
VAS: Visual analog scale
